# Analysis of the Expression and Polymorphism of *APOE, HSP, BDNF,* and *GRIN2B* Genes Associated with the Neurodegeneration Process in the Pathogenesis of Primary Open Angle Glaucoma

**DOI:** 10.1155/2015/258281

**Published:** 2015-03-29

**Authors:** Alicja Nowak, Ireneusz Majsterek, Karolina Przybyłowska-Sygut, Dariusz Pytel, Katarzyna Szymanek, Jerzy Szaflik, Jacek P. Szaflik

**Affiliations:** ^1^Department of Clinical Chemistry and Biochemistry, Medical University of Lodz, Hallera 1 Square, 90-647 Lodz, Poland; ^2^The Abramson Family Cancer Research Institute, Department of Cancer Biology, University of Pennsylvania, Perelman School of Medicine, University of Pennsylvania, Philadelphia, PA 19104, USA; ^3^Department of Biochemistry and Molecular Biology, Hollings Cancer Center, Medical University of South Carolina, Charleston, SC 29425, USA; ^4^Department of Ophthalmology, Medical University of Warsaw, Sierakowskiego 13 street, 03-709 Warsaw, Poland; ^5^SPKSO Ophthalmic Hospital, Sierakowskiego 13 street, 03-709 Warsaw, Poland

## Abstract

Glaucoma is characterized by optic neuropathy of the RGC or retinal nerve fiber. The aim of this study was to evaluate a relationship between the neurodegenerative genes' polymorphisms of the *APOE* (rs449647), *BDNF* (rs2030324), *GRIN2B* (rs3764028), and *HSP70-1* (rs1043618) and the occurrence risk of POAG and to investigate its effect on allele-specific gene expression. Genomic DNA was extracted from peripheral blood. Analysis of the genes' polymorphisms was performed using PCR-RFLP. The level of mRNA expression was determined by QRT-PCR. We showed a statistically significant association of *BDNF* and *APOE* genes' polymorphisms with a risk of POAG occurrence. There was a statistically significant association of the rs2030324 polymorphism with progression of POAG based on cup disc ratio value and rs1043618 polymorphism based on nerve fiber index and rim area. Furthermore, we found that mean *HSP70-1* mRNA expression was significantly lower in the case of individuals with the G/G genotype than in the case of minor allele carriers, that is, G/C and C/C. We also found that *BDNF* and *HSP70-1* expression level are associated with the progression of POAG based on rim area value. In conclusion, our results suggest that *BDNF*, *APOE*, and *HSP70-1* genes might be associated with a risk of POAG occurrence in the Polish population.

## 1. Introduction

Glaucoma is one of the causes of blindness in the world, especially among the elderly [1]. The recent data have indicated that the number of people suffering from glaucoma reaches nearly 70 million, and among them, more than 7 million people are blind. The most common type of glaucoma is primary open angle glaucoma (POAG). In Poland, it constitutes about 80% of patients with glaucoma [2, 3].

Glaucoma is an optic neuropathy characterized by retinal ganglion cell (RGC) death, axon loss, and an excavated appearance to the optic nerve head [4]. Elevated intraocular pressure (IOP) is one of the causes of glaucoma development. However, in some patients with glaucoma, IOP is observed within normal limits (11–21 mmHg) [5]. On the other hand, despite the reduction of IOP as a result of medical or surgical treatment, progressive loss of vision is still noticeable [6]. For this reason, there must be an IOP-independent mechanism leading to glaucomatous degeneration. Glaucoma along with Alzheimer's (AD), Parkinson's disease (PD), and multiple sclerosis (MS) is classified as a neurodegenerative disorder. A lot of data have indicated that there are similarities in cellular events leading to the development of glaucoma in the aforementioned diseases. These similarities include the selective loss of neuron populations, transsynaptic degeneration in which the disease spreads from injured neurons to connected neurons, and common mechanisms of cell injury and death [6]. Among patients with AD and PD there exists higher morbidity of glaucoma [7]; moreover optic nerves from AD patients are characterized by the loss of RGCs, the earliest dying cells in glaucoma [8]. In another study, it was reported that, in patients with MS, reduction of the retinal nerve fiber layer (RNFL) thickness is observed [9], while loss of RGCs in glaucoma is caused by RNFL thinning [10].

The underlying cause of RGC death in neurodegenerative diseases seems to be apoptosis, a programmed cell death [11–13]. Processes that lead to apoptosis in the development of glaucoma include blockage of axonal transport [14], glutamate excitotoxicity [15], antibodies to heat shock proteins [16], and ischemia [17]. Normal axonal transport is vital to the healthy functioning of neurons, whereas retrograde transport of neurotrophins may be necessary for the survival of retinal ganglion cells. One of the neurotrophins which plays a role in retrograde axonal transport is brain-derived neurotrophic factor (BDNF). BDNF is essential to the growth and survival of nerve cells [18]. Excitotoxicity may also be associated with apoptosis in RGCs [15]. Excitotoxicity is the pathological process which leads to damage and consequently to the death of neurons by the overactivation of receptors for the excitatory neurotransmitter glutamate, such as the NMDA receptor (N-methyl-d-aspartate). Overactivation of the NMDA receptor causes enhanced influx of ions into the cell, especially Ca^2+^. Excessive influx of calcium activates enzymes that degrade cell membranes, cellular proteins, and nucleic acids, which finally leads to apoptosis [19]. It was observed that the level of glutamate in the vitreous was increased in glaucomatous patients and animals with experimentally induced glaucoma [15]. These data may suggest participation of excitotoxicity in RGC apoptosis. In the development of glaucoma, HSPs and antibodies against them may also be involved [20, 21]. Damage to the optic nerve and related various stress factors may lead to an overexpression of HSPs. Tezel et al. (2000) have demonstrated an increase in immunostaining of HSPs in the glaucomatous eyes [20]. Meanwhile, Tezel et al. (2004) have also underlined the role of HSPs in the pathological development of glaucoma by the activation of the autostimulatory response resulting in degeneration of the optic nerve [21]. Another protein that may influence the development of glaucoma is apolipoprotein E (APOE). APOE belongs to the class of lipoproteins that regulate the metabolism of lipids in the body. It is widely expressed in various tissues of the body but the highest expression is observed in the liver and brain. In the CNS, APOE is involved in homeostasis of cholesterol, which plays an important role in myelin production, function, and integrity. Disturbances in the cholesterol metabolism have been associated with ageing and the development of neurodegenerative diseases, such as AD [22–24]. Several studies have suggested that those who inherit the* APOEε4* gene may be predisposed to development of some disorders including atherosclerosis and AD [22, 25]. Furthermore,* APOEε4* may be responsible for disrupting the A*β* clearance and therefore the accumulation of the toxic amyloid plaque in the brain [26].

In our study, we examine the relationship between single nucleotide polymorphisms (SNPs) of* APOE* (rs449647),* BDNF* (rs2030324)*, GRIN2B* (rs3764028), and* HSP70-1* (rs1043618) genes and the possible occurrence risk of primary open angle glaucoma in the Polish population. Additionally, we tested the effect of these SNPs on allele-specific mRNA expression and correlated the results we obtained with known clinical parameters.

## 2. Materials and Methods

### 2.1. Study Subject

The case-control study included a total of 769 nonfamilially related Caucasian subjects. The subjects with POAG consisted of 363 patients (98 males and 265 females; mean age 73 ± 10), while the control group, without glaucoma symptoms, consisted of 406 patients (174 males and 232 females; mean age 68 ± 13) (Table 1). All patients and controls were age-matched (no difference was calculated, *P* > 0.05). All subjects underwent ophthalmologic examinations, including evaluation of intraocular pressure, best-corrected visual acuity, slit-lamp examination, gonioscopy, and fundus examination using noncontact and contact fundus lenses with a slit lamp. Inclusion criteria for the control group were as follows: intraocular pressure within normal limits, no changes in the optic nerve after examination of the fundus, and no changes in the structures of the anterior and posterior segment of the eye after slit-lamp examination. If any one or more of these conditions were not fulfilled, those subjects were excluded from the control group.

Among the glaucomatous patients, the diagnosis of POAG was stated prior to enrollment and in accordance with the guidelines of European Glaucoma Society (Terminology and Guidelines for Glaucoma, Second Edition, Dogma, Savona 2003, Italy). A comprehensive medical history was obtained from each glaucomatous patient. The following conditions excluded patients from the study group: use of any eye drops other than antiglaucoma preparations, present or past treatment with glucocorticosteroids or immunosuppressive therapy (if these treatments had not been stopped at least 1 year before collection of specimens), and any ocular surgeries or laser treatments performed in the previous 6 months.

All subjects involved in the study were nonfamilially related Caucasians and inhabited the Warsaw District in Poland. All patients and controls were examined in the Department of Ophthalmology, Medical University of Warsaw (Poland). The study design was approved by the Committee for Bioethics of the Medical University of Lodz (Poland) and met the tenets of the Declaration of Helsinki. An informed written consent was explained and signed by all participants before the study was initiated.

### 2.2. DNA Preparation and Genotyping

Blood samples were collected in 5 mL EDTA tubes. Genomic DNA was isolated from the white blood cells using the kit QIAamp DNA Blood Mini Kit (Qiagen, Chatsworth, CA, USA), according to the manufacturer's instructions. DNA purity and concentrations were determined spectrophotometrically at 260 and 280 nm. Each DNA sample was stored at −20°C until analysis was performed.

The polymerase chain reaction-restriction fragment length polymorphism (PCR-RFLP) method was used to determine the genotypes of* BDNF*,* HSP70-1*,* GRIN2B,* and* APOE* genes according to previously described procedures with some modifications. Primer sequences used for the amplification are the same as described in previous publications [27–31]. PCR assay was performed in a total reaction volume of 20 *μ*L containing the following components: 10 ng genomic DNA, 1.25 U Taq polymerase (Qiagen, Chatsworth, CA, USA) in 1x PCR buffer (100 Mm Tris-HCl, pH 8.3; 500 mM KCl; 11 mM MgCl_2_, 0.1% gelatine), 1.5 mM MgCl_2_, 50 nM dNTPs, and 250 nM of each primer (Sigma-Aldrich, St. Louis, MO, USA). Thermal cycling conditions with primer sequences are displayed in Table 2. The PCR was carried out in a T100 thermal cycler (Bio-Rad, Richmond, CA, USA). To examine the rs449647 polymorphism, the 1423 bp fragment was amplified using PCR reaction. Then, the 1423 bp product was used as a template for the following RFLP-PCR analyses: the 227 bp fragment, generated by amplification with mismatched primers, was digested for 16 hours at 37°C with 2 U of the restriction enzyme* Dra*I (New England Biolabs, Ipswich, MA, USA). The fragment of the* APOE* gene containing A variant was digested into the 206 bp and 21 bp fragments whereas the T remained intact. To genotype the rs3764028 polymorphism, PCR amplification product, 115 bp, was digested with 1 U* Hpy*CH4IV (New England Biolabs, Ipswich, MA, USA) for 16 hours at 37°C. The wild-type C allele remained uncut, while the A allele was digested into 96 bp and 19 bp fragments. To examine the rs1043618 polymorphism, PCR amplification product, 488 bp, was digested with 1 U* Bsr*BI (New England Biolabs, Ipswich, MA, USA) for 16 hours at 37°C. The wild-type G allele was digested into 461 bp and 27 bp fragments, while the C allele, which lacks the restriction site, remained uncut. The genotype of the rs2030324 polymorphism was obtained from the 348 bp fragment, which was digested for 16 hours at 37°C with 1 U of the restriction enzyme* Hpy*CH4IV (New England Biolabs, Ipswich, MA, USA). The fragment of the* BDNF* gene containing C variant was digested into 268 bp and 79 bp fragments and the fragment containing T remained intact. Digested PCR products were separated by electrophoresis on a 3% agarose gel in a TAE buffer and visualized by ethidium bromide staining under UV light. In the study, to verify the activity of the restriction enzymes, we also used pBR322 plasmid (Invitrogen, Carlsbad, CA, USA) with multiple cloning sites containing 2, 10, and 3 restriction sites for* Bsr*BI,* Hpy*CH4IV, and* Dra*I enzymes, respectively.

### 2.3. RNA Isolation and cDNA Synthesis

Blood samples were collected in 5 mL EDTA tubes. Total RNA was isolated from peripheral lymphocytes using Fenozol reagent (A&A Biotechnology, Gdynia, Poland) according to the manufacturer's protocol. Total RNA was extracted from 54 subjects with POAG and 49 subjects without glaucoma. RNA was eluted in 50 *μ*L RNase-free water and stored at −20°C. The yields were quantified spectrophotometrically. RNA with a 260/280 nm ratio in the range of 1.8–2.0 was considered high quality and was used for further analysis. First-strand cDNA was synthesized by reverse transcription of 1 *μ*g of total RNA using AffinityScript QPCR cDNA Synthesis Kit (Agilent Technologies, Santa Clara, CA, USA) following the manufacturer's protocol.

### 2.4. Real-Time Quantitative PCR

For real-time PCR analysis of* APOE*,* BDNF*,* GRIN2B*, and* HSP70-1* mRNA, Brilliant II SYBR QPCR and QRT-PCR Master Mix Kits (Agilent Technologies, Santa Clara, CA, USA) were used according to the manufacturer's instruction. The* GAPDH* gene was used as the internal sample control. The sequences of specific primers for* GAPDH* and target genes are presented in Table 3. For real-time quantitative PCR (RT-qPCR), reaction mixtures consisting of 400 nM each primer, 2x Brilliant II SYBR Green QPCR master mix, and 20 ng of template cDNA were preincubated for 10 minutes at 95°C followed by 40 cycles of 95°C for 30 sec, 60°C for 30 sec, and 72°C for 30 sec. The reactions were performed in duplicate. The RT-qPCR reaction was carried out using the Mx3005P from Agilent Technologies.

A positive result was defined by a threshold cycle (Ct) value lower than 40 (the Ct value is determined by the number of cycles needed to exceed the background signal). Abundance of target genes mRNA in studied material was quantified by the ΔCt method.

### 2.5. Statistical Data Analysis

To compare the distributions of demographic variables and selected risk factors between patients and controls, the Chi-square test was used. The observed number of cases for each genotype in the study and control group was compared with the expected number according to the Hardy-Weinberg principle, using the *χ* ^2^ test. The *χ* ^2^ analysis was also used to test the significance of the differences between distributions of genotypes in glaucoma patients and controls. The association between case-control status and each polymorphism, measured by the odds ratio (OR) and its corresponding 95% confidence interval (CI), was estimated using an unconditional multiple logistic regression model. When calculating the probability, Pearson correction was used, and if the expected cell values were less than 5, Fisher's exact test was used. The odds ratios were then adjusted for possible interfering factors, including hypertension, low blood pressure, vascular disease, and diabetes. Clinical features in patients with POAG were also compared between genotypes of each polymorphism by using an analysis of variance (ANOVA with* post hoc* Tukey's HSD test) for the comparison with discrete variables. The nonparametric Mann-Whitney *U* test was applied to determine the levels of mRNA expression in blood of POAG patients and healthy subjects. The nonparametrical statistical tests (ANOVA with* post hoc* Tukey's HSD test) were applied to compare the level of mRNA expression with genotypes of each polymorphism in studied material specimens. A *P* value of less than 0.05 was considered statistically significant. Significant probability values obtained were analyzed for multiple testing using Bonferroni correction (*P* value after Bonferroni correction (*P*_corr_)). Post hoc power analysis with preestablished effect size, error probability, and sample size was performed using the G^*^Power version 3.1.3 program [32]. Statistical analysis was performed using STATISTICA 6.0 software (Statsoft, Tulsa, OK, USA).

## 3. Results 

The genotype and allele frequency and the odds ratios of the examined genes' polymorphisms in the study and control group are displayed in Table 4. The observed genotype frequencies of* HSP70-1* (*P* > 0.05; *χ* ^2^ = 2.56),* BDNF* (*P* > 0.05; *χ* ^2^ = 0.37),* GRIN2B* (*P* > 0.05; *χ* ^2^ = 1.46), and* APOE* (*P* > 0.05; *χ* ^2^ = 3.51) in the control group were in agreement with Hardy-Weinberg equilibrium.

A statistically significant increase in the frequency of the C/T* BDNF* genotype (OR 1.72; 95% CI, 1.23–2.39; *P* < 0.001) and T/T* BDNF* genotype (OR 2.06; 95% CI, 1.36–3.12; *P* < 0.001) as well as T* BDNF* allele (OR 1.45; 95% CI, 1.19–1.78; *P* < 0.001) in patients with POAG in comparison with control group. And after the Bonferroni correction, the positive association remained (*P*_corr_ < 0.001). We also observed positive association of the −491 T* APOE* allele (OR 1.29; 95% CI, 1.02–1.63; *P* = 0.034) occurrence with an increased POAG development risk but after using Bonferroni correction, this association was no longer statistically significant (*P*_corr_ = 0.136). However, comparison of the genotype and allele distributions of the −421 C/A* GRIN2B* gene polymorphism as well as 190 G/C polymorphism of* HSP70-1* gene and analysis of the odds ratio (OR) showed no statistically significant differences between POAG patients and controls (*P* > 0.05). Using power calculation we demonstrated that the study had >90% power in detecting associations of rs449647 and rs2030324 polymorphisms with risk of POAG, at a significance level of 0.05 (df = 2). We were not able to show an association between the rs3764028 polymorphism and POAG in our population, which possibly may be due to a lack of power calculation. In addition, statistical analysis demonstrated that the polymorphisms of* APOE*,* BDNF*,* GRIN2B,* and* HSP70-1* genes are not related with hypertension, diabetes, low blood pressure, and vascular disease.

Analyses of the genes' polymorphisms in correlation with the clinical parameters in POAG patients for each eye counted separately are displayed in Tables 5–8. There was statistically significant association of the* BDNF* (rs2030324) gene polymorphism with progression of POAG based on the cup disc ratio value – c/d, *P* = 0.004  (*P*_corr_ = 0.016). We found an association of the T/T genotype of* BDNF* gene with a decreased c/d ratio value in patients with POAG group (Table 5). Moreover, the analysis of the* HSP70-1* (rs1043618) gene polymorphism showed a correlation with progression of POAG based on clinical parameters: nerve fiber index (NFI) and rim area (RA), *P* = 0.004  (*P*_corr_ = 0.016) and *P* = 0.009  (*P*_corr_ = 0.036); respectively. We observed an association of a decreased RA value (rim area) with the occurrence of the 190 C/C genotype of* HSP70-1* gene (Tukey's HSD test: *P* < 0.05; RA C/C versus G/C). An increased NFI value was observed in patients with POAG group connected with the 190 C/C* HSP70-1* genotype (Tukey's HSD test: *P* < 0.05; NFI C/C versus G/C) (Table 6). However, there were no statistically significant associations of* GRIN2B* (rs3764028) and* APOE* (rs449647) genes' polymorphisms with progression of POAG based on clinical parameters, *P* > 0.05 (Tables 7 and 8, resp.).

**Figure 1:**
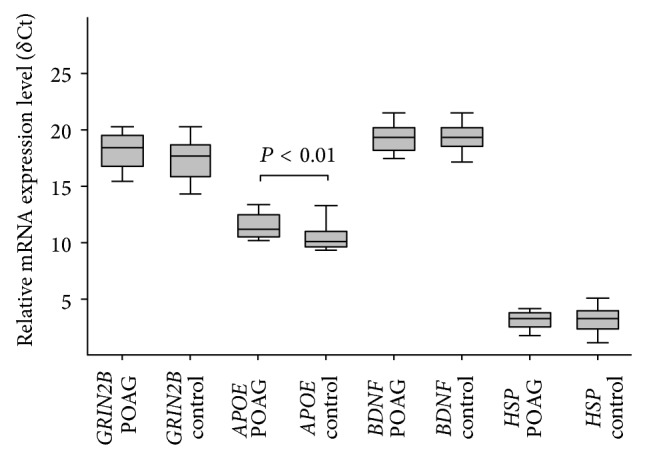
Comparison of mRNA expression level of* GRIN2B*,* APOE*,* BDNF,* and* HSP70-1* genes in the blood between primary open angle glaucoma (POAG) and control groups measured by real-time PCR. Error bars represent the means ± SEM; *U* test, *P* < 0.01.

The results of* APOE*,* BDNF*,* GRIN2B,* and* HSP70-1* genes' expression in blood of POAG patients and healthy subjects are presented in Figure 1. The* APOE* gene displayed a significantly higher expression in the blood of POAG patients than in healthy subjects (*P* = 0.01  (*P*_corr_ = 0.04)). No statistically significant differences were found in* BDNF*,* GRIN2B,* and* HSP70-1* mRNA levels between POAG patients and controls (*P* > 0.05).

Distributions of genotypes and allele frequencies of genes' polymorphisms with regard to levels of mRNA expression are presented in Table 9. It was found that mean* HSP70-1* mRNA expression was significantly lower in the cases of individuals with the G/G genotype than in the cases of minor allele carriers, that is, G/C heterozygotes and C/C homozygotes (ANOVA *P* = 0.016; G/G versus G/C *P* < 0.05 and G/G versus C/C *P* < 0.05). However, after using Bonferroni correction, this *P* value was no longer statistically significant (*P*_corr_ = 0.064). Any statistically significant association of* BDNF*,* APOE*, and* GRIN2B* genes' polymorphisms in POAG patients with mRNA expression levels was observed.

Analyses of the genes' expression levels in correlation with the clinical parameters in POAG patients for each eye counted separately are displayed in Tables 10–13. There was statistically significant association of the* BDNF* expression level with progression of POAG based on RA value, *P* = 0.011  (*P*_corr_ = 0.044). We observed a decrease of* BDNF* expression level with the advancement of glaucoma (Table 10). Furthermore, the analysis of the* HSP70-1* expression level showed a correlation with progression of POAG based on RA value, *P* = 0.004  (*P*_corr_ = 0.016). We found an increase of* HSP70-1* expression level with a decrease of the RA parameter value (Table 11). There were no statistically significant associations of the* GRIN2B* and* APOE* expression level with progression of POAG based on clinical parameters, *P* > 0.05 (Tables 12 and 13, resp.).

## 4. Discussion

Glaucoma is a disease characterized by degeneration of optic nerve axons and death of retinal ganglion cells and is frequently associated with high IOP. The progressive loss of vision often continues despite antihypertensive treatment; hence IOP-independent mechanisms are also implicated in glaucomatous degeneration [6]. However, many of the factors that cause degeneration of the optic nerve have not yet been known. Apoptotic RGC death is one of the hypotheses explaining the mechanism of glaucoma development. Typical apoptotic changes were observed in RGCs, including DNA degradation and blebbing of the plasma membrane [33, 34]. One of the mechanisms leading to apoptosis of RGCs is excitotoxicity [15]. In our research, we focused on determining the relationship between the −421 C/A polymorphism of* GRIN2B* gene and the risk development of POAG.* GRIN2B* gene encodes the NR2B subunit of the NMDA receptor, which is responsible for the attachment of glutamate [28]. However, no significant differences in the distribution of genotypes and alleles were observed between the study group and the control group (*P* > 0.05). We could not compare the obtained results with other outcomes relating to the relationship of* GRIN2B* gene polymorphism with the risk of POAG because of the lack of data in the literature. In turn, study with Alzheimer's patients has shown statistically significant differences in genotype (*P* = 0.029) and allele (*P* = 0.010) frequencies for −421 C/A* GRIN2B* in comparison with the control group [28]. Jiang and Jia (2009) using a luciferase assay have demonstrated a 34.69–39.79% decrease in transcriptional activity for −421 C allele of* GRIN2B* promoter compared with −421 A allele. These data have shown that −421 C allele can affect the decreased transcriptional activity of the* GRIN2B* gene and thereby reduce the expression of the NR2B subunit.* GRIN2B* decreased expression can be associated with the presence of the putative zinc finger binding sites, the ras-responsive element binding protein (RREB) within the −421 C/A polymorphism [28]. The study has found that RREB may have negative transcription factors and reduce the promoter activity of some genes and consequently inhibit the protein expression [35]. However, our study showed no statistically significant association between* GRIN2B* gene polymorphism in POAG patients and mRNA expression levels (*P* > 0.05).

Suppression of neurotrophic factors can also lead to nerve cell death. Yang et al. (2012) have found that the mRNA transcription level of p53 was elevated in BDNF knocked down MDA-MB-231 cells compared with vector control [36]. An increase of p53 transcription induced by lower BDNF expression may enhance cell apoptosis, since p53 is a well-known proapoptotic protein [37]. BDNF has been found to mediate protection from apoptosis by p53 activation [38]. Numerous studies have indicated that the cell death of RGCs in glaucoma may be associated with a deficit of neurotrophins, including BDNF. Quigley et al. (2000) have shown that, in rats with experimentally induced glaucoma, BDNF flow to the retina is significantly reduced [39], whereas, in another study, using an animal model of glaucoma, it was demonstrated that injection of BDNF into the vitreous cavity is associated with greater RGC survival than untreated eyes [40]. Recent study has confirmed the participation of BDNF in maintaining the inner retinal integrity under normal conditions and adverse effects of neurotrophin scarcity on the retina and the optic nerve during development of glaucoma. BDNF(+/−) animals showed greater susceptibility to morphological, functional, and molecular degenerative changes in the retina caused by elevated IOP [41]. Our study showed no significant differences in* BDNF* mRNA levels in the blood between POAG patients and controls (*P* > 0.05), while analysis of the* BDNF* expression level in correlation with the clinical parameters showed a decrease of mRNA levels with a decrease of the RA value (*P* = 0.011), which confirms the protective role of BDNF in the development of glaucoma. Gao et al. (1997) have observed elevated mRNA expression of* BDNF* after optic nerve injury in animal models [42]. Using the northern blot assay, they demonstrated a 38% elevation in BDNF expression above control levels 48 hours after optic nerve injury. Our analysis of the genotype and allele frequency of* BDNF* (rs2030324) gene polymorphism showed a statistically significant increase in the frequency of the C/T* BDNF* genotype (OR 1.72; 95% CI, 1.23–2.39; *P* < 0.001) and T/T* BDNF* genotype (OR 2.06; 95% CI, 1.36–3.12; *P* < 0.001) as well as T* BDNF* allele (OR 1.45; 95% CI, 1.19–1.78; *P* < 0.001) in POAG patients in comparison with healthy controls. Interestingly, our results showed the increase in cup disc ratio value associated with the T/T* BDNF* genotype (_ANOVA_ *P* = 0.004; Tukey's HSD test: *P* < 0.05; T/T versus C/T). Thus, the mechanism of glaucoma development according to the* BDNF* (rs2030324) polymorphism remains unclear. The data suggest that the* BDNF* (rs2030324) polymorphism may be considered a risk factor for POAG occurrence, which is not associated with its progression. However, there is a lack of literature data on the rs2030324 gene polymorphism association with the risk development of POAG, whereas, in the study performed by Vepsäläinen et al. (2005) it was demonstrated that there was no relationship of this polymorphism with risk occurrence of AD [43]. The rs2030324 polymorphism is located in the promoter site of the gene; hence it might have an influence on gene expression [44]. However, our research showed no statistically significant association of* BDNF* genes polymorphism in POAG patients with mRNA expression levels (*P* > 0.05).

Recently, a lot of data have demonstrated that HSPs are also involved in the neuropathy of glaucoma. HSPs may have a pathogenic or protective effect in the development of glaucoma. A variety of stress conditions, including ischemia and excitotoxicity, can lead to overexpression of HSPs [20, 21]. Chidlow et al. (2014) have indicated that HSP70 and HSP27 expression was induced in the retina and optic nerve in four discrete models of RGC degeneration: axonal injury, somatodendritic injury, chronic hypoperfusion, and experimental glaucoma [45]. Meanwhile, Kwong et al. (2015) have demonstrated that pharmacological induction of HSP70 expression has a beneficial effect on the survival of injured RGCs. They have demonstrated that the overexpression of HSP70 in the retina was approximately twofold higher compared with expression in control animals without any treatment [46]. The 190 G/C polymorphism, which is located in the 5′UTR region, can affect the level of HSP expression. A functional study of this polymorphism has reported that the C allele is related to reduced promoter activity and a lower level of HSP70 protein, compared to the G allele [47]. The above data highlight the hypothesis that the decline of HSP levels leads to a reduction in their protective role in glaucoma pathogenesis. Our study demonstrated that mean* HSP70-1* mRNA expression was significantly lower in the cases of individuals with the G/G genotype than in cases of minor allele carriers, that is, G/C heterozygotes and C/C homozygotes (*P* < 0.05). In turn, this finding highlights the participation of HSPs in the pathogenesis of glaucoma by an autoimmune response. Moreover, analysis of the* HSP70-1* expression level in correlation with the clinical parameters showed an increase of mRNA levels with a decrease of RA value (*P* = 0.004) and consequently with the progression of POAG. Comparison of genotypes and allele distributions of the 190 G/C* HSP70-1* gene polymorphism showed no statistically significant differences between POAG patients and controls (*P* > 0.05). Likewise, Tosaka et al. (2007) have not found any relationship between POAG and the 190 G/C polymorphism, but they have indicated that another polymorphic variant (−110 A/C) in* HSP70-1* gene is associated with development of glaucoma (AA versus AC + CC; *P* = 0.007) [48]. Our data also demonstrated the relationship of 190 G/C* HSP70-1* gene polymorphism with a progression of POAG based on NRI and RA clinical parameters (*P* = 0.004, *P* = 0.009, resp.). We observed an association of decreased RA value (rim area) and increased NFI value with the occurrence of the 190 C/C genotype of* HSP70-1* gene (Tukey's HSD test: *P* < 0.05; RA C/C versus G/C) confirming the role of 190 C/C genotype in the progression of POAG.

The major risk factor for neurodegenerative diseases, including glaucoma, seems to be APOE. Research has shown that APOE is synthesized by Müller cells in the retina and secreted into the vitreous. Subsequently, the apolipoprotein E gets to the RGCs and is transported along the optic nerve, which may play a role in axonal nutrition [49]. It has also been suggested that the APOE isoform may be related to neuronal degeneration in glaucoma. Vickers et al. (2002) have shown that inheritance of the* APOEε4* allele may represent a risk factor for glaucoma, particularly for cases associated with normal intraocular pressures [50]. It is demonstrated that in Alzheimer's disease APOE*ε*4 interacts with *β*-amyloid, resulting in a decreased A*β* clearance, which leads to the accumulation of toxic amyloid plaques in the brain [26]. It was observed, in animal models with glaucoma, that *β*-amyloid is also formed in RGC [51]. Using a real-time PCR assay, we demonstrated that* APOE* gene had a significantly higher expression in the blood of POAG patients than in healthy subjects (*P* < 0.01), which suggests its pathological role in the development of POAG. Moreover, our study showed that the* APOE* −491 T allele was more frequent in POAG patients compared to healthy subjects (26% versus 21%; *P* = 0.034). However, other studies have shown that the interaction of the −491 T allele with MYOC gene promoter (−1000 G) can lead to IOP increase and thereby the degeneration of the optic nerve [52]. Compared to our findings, mRNA expression in the blood of POAG with genotype of −491 A/T* APOE* gene polymorphism did not show any statistical significance (*P* > 0.05).

## 5. Conclusion

In conclusion, our results suggest that* BDNF* (rs2030324) and* APOE* (rs449647) genes' polymorphisms might be associated with a risk of POAG occurrence in the Polish population. Moreover, our findings indicate that* HSP70-1* (rs1043618) gene polymorphism can affect the expression of* HSP70-1*. These data might be useful in the better understanding of POAG etiology. Identification of SNPs associated with glaucoma may be helpful in the discovery of molecular markers, which might allow for a fast diagnosis, more effective treatment, and a better prognosis for patients suffering from glaucoma.

## Figures and Tables

**Table 1:** The clinical parameters characteristic of open angle glaucoma (POAG) patients and control groups.

	Parameters	Patient groups *n* = 363	Control groups *n* = 406
Number	Gender (male/female)	98/265	174/232
	Hypertension^∗^	218	132
	Low blood pressure^∗∗^	75	54
	Vascular disease	124	92
	Diabetes mellitus type 2	70	104

Mean ± SD	Age (years)	73 ± 10	64 ± 16
	Intraocular pressure, IOP (mmHg)	12.82 ± 2.9	11.9 ± 1.9
	Cup disk ratio (c/d) right eye/left eye	0.74 ± 0.15/0.74 ± 0.27	PNM
	Rim area (RA) right eye/left eye	1.45 ± 3.57/1.29 ± 1.07	PNM
	Retinal nerve fiber layer (RNFL) right eye/left eye	0.39 ± 3.25/0.94 ± 12.13	PNM

** **	Nerve fiber index (NRI) right eye/left eye	32.47 ± 19.57/25.92 ± 17.11	PNM

^∗^Systolic pressure >140; diastolic pressure >90 mmHg.

^∗∗^Systolic pressure <90; diastolic pressure <60 mmHg.

PNM: parameter not measured.

**Table 2:** Primer sequences and restriction endonucleases used in the gene polymorphisms analysis by polymerase chain reaction-restriction fragment length polymorphism (PCR-RFLP).

Gene polymorphism	Primer sequence	Annealing [°C]	PCR product [bp]	Enzyme
rs1043618	F: 5′-CGCCATGGAGACCAACACCC-3′ R: 5′-GCGGTTCCCTGCTCTCTGTC-3′	63	488	*Bsr*BI

rs3764028	F: 5′-CGCTCTCCGTCGGTGCTGTT-3′ R: 5′-CTGGGGAAGTGGGGTGGTAACG-3′	61	115	*Hpy*CH4IV

rs2030324	F: 5′-TTGCACATCCTGCTCAAGTC-3′ R: 5′-TTGCTAGGAGAAAAGCCATGA-3′	62	348	*Hpy*CH4IV

rs449647	F: 5′-CAAGGTCACACAGCTGGCAAC-3′ R: 5′-TCCAATCGACGGCTAGCTACC-3′	69	1426	—
	F (mismatched): 5′-TGTTGGCCAGGCTGGT**T**T**T**AA-3′ R: 5′-CTTCCTTTCCTGACCCTGTCC-3′	63	227	*Dra*I

**Table 3:** Primer sequences used in QRT-PCR analysis.

Gene	Primer sequence	PCR product [bp]
*GAPDH *	F: 5′-CACCTTCCCCATGGTGTCT-3′ R: 5′-CCCCGGTTTCTATAAATTGAGC-3′	120

*BDNF *	F: 5′-CAAATACAAGACCCTGCTTGTG-3′ R: 5′-CCACCATTTTTGCACTTGCTT-3′	90

*APOE *	F: 5′-TGGACAAGTCTGGGATCCTT-3′ R: 5′-CATCTTCCTGCCTGTGATTG-3′	78

*HSP70-1 *	F: 5′-GGGAAGCCTTGGGACAAC-3′ R: 5′-TGATTGGCTCAGAAGGGAAA-3′	80

*GRIN2B *	F: 5′-AGCAATGGGACTGTCTCACC-3′ R: 5′-AACATCATCACCCATACGTCAG-3′	85

**Table 4:** The genotype and allele frequency and odds ratios (OR) of the gene polymorphisms in open angle glaucoma (POAG) patients and controls.

Genotype or allele	POAG patients *n* = 363	Control subjects *n* = 406	OR (95% CI)	*P*	OR adjusted^1^ (95% CI)	*P*
***HSP70-1***						
G/G	155 (43%)	179 (44%)	Ref	Ref	Ref	Ref
G/C	169 (47%)	170 (42%)	1.15 (0.85–1.55)	0.371	1.29 (0.94–1.77)	0.107
C/C	39 (10%)	57 (14%)	0.79 (0.50–1.25)	0.315	0.67 (0.42–1.08)	0.103
G	479 (66%)	528 (65%)	Ref	Ref	Ref	Ref
C	247 (34%)	284 (35%)	0.96 (0.78–1.18)	0.699	0.94 (0.76–1.17)	0.630

Post hoc power analysis: df = 2, *n* = 769 Effect size *w* = 0.132; power (1 − *β* err prob) = 0.919						

***BDNF***						
C/C	86 (24%)	146 (36%)	Ref	Ref	Ref	Ref
**C/T**	192 (53%)	190 (47%)	**1.72 (1.23**–**2.39)**	**<0.001**	**1.43 (1.04**–**1.95)**	**0.024**
**T/T**	85 (23%)	70 (17%)	**2.06 (1.36**–**3.12)**	**<0.001**	**2.01 (1.92**–**2.23)**	**0.008**
C	364 (50%)	482 (59%)	Ref	Ref	Ref	Ref
**T**	362 (50%)	330 (41%)	**1.45 (1.19**–**1.78)**	**<0.001**	**1.45 (1.18**–**1.78)**	**<0.001**

Post hoc power analysis: df = 2, *n* = 769 Effect size *w* = 0.262; power (1 − *β* err prob) = 0.999						

***GRIN2B***						
C/C	331 (91%)	360 (89%)	Ref	Ref	Ref	Ref
C/A	31 (8%)	46 (11%)	0.73 (0.45–1.18)	0.203	0.80 (0.47–1.35)	0.417
A/A	1 (1%)	0 (0%)	—	—	—	—
C	693 (95%)	766 (94%)	Ref	Ref	Ref	Ref
A	33 (5%)	46 (6%)	0.79 (0.50–1.25)	0.320	0.87 (0.52–1.43)	0.589

Post hoc power analysis: df = 2, *n* = 769 Effect size *w* = 0.096; power (1 − *β* err prob) = 0.660						

***APOE***						
A/A	205 (56%)	257 (63%)	Ref	Ref	Ref	Ref
A/T	127 (35%)	124 (31%)	1.28(0.94–1.75)	0.112	1.19 (0.85–1.65)	0.302
T/T	31 (9%)	25 (6%)	1.55 (0.89–2.72)	0.119	1.61 (0.88–2.93)	0.115
A	537 (74%)	638 (79%)	Ref	Ref	Ref	Ref
**T**	189 (26%)	174 (21%)	**1.29 (1.02**–**1.63)**	**0.034**	**1.35 (1.05**–**1.73)**	**0.018**

Post hoc power analysis: df = 2, *n* = 769 Effect size *w* = 0.167; power (1 − *β* err prob) = 0.990						

^1^Odds ratio adjusted for hypertension, low blood pressure, vascular disease, and diabetes.

**Table 5:** Analysis of *BDNF* (rs2030324) gene polymorphism depending on the clinical parameters in patients with primary open angle glaucoma (POAG) for each eye counted.

Clinical parameter	Genotype	POAG patients	Mean	SD	Quartile 25%	Median	Quartile 75%	*P*
NFI	C/C	87	28.2	20.1	15.0	23.0	38.0	0.098
	C/T	231	33.6	23.3	17.0	26.0	42.0	
	T/T	84	24.9	12.9	18.0	23.0	29.0	

**c/d**	C/C	155	0.74	0.13	0.67	0.70	0.85	**0.004**
	C/T	364	0.74	0.16	0.60	0.80	0.85	
	T/T	150	0.66^#^	0.16	0.60	0.70	0.80	

RA	C/C	121	1.21	0.37	0.96	1.22	1.42	0.092
	C/T	276	1.23	0.43	0.91	1.26	1.49	
	T/T	125	1.30	0.35	1.03	1.29	1.56	

RNFL	C/C	121	0.18	0.08	0.13	0.19	0.23	0.551
	C/T	276	0.21	0.20	0.12	0.19	0.26	
	T/T	124	0.21	0.16	0.14	0.19	0.26	

NFI: nerve fiber index; c/d: cup disc ratio; RA: rim area; RNFL: retinal nerve fiber layer.

Tukey's HSD test: *P* < 0.05 (^#^C/T versus T/T).

**Table 6:** Analysis of *HSP70-1* (rs1043618) gene polymorphism depending on the clinical parameters in patients with primary open angle glaucoma (POAG) for each eye counted.

Clinical parameter	Genotype	POAG patients	Mean	SD	Quartile 25%	Median	Quartile 75%	*P*
**NFI**	G/G	178	31.6	21.3	16.0	25.5	43.0	**0.004**
	G/C	183	26.3	17.8	16.0	23.0	29.0	
	C/C	41	44.16^#^	25.1	22.0	26.0	59.5	

c/d	G/G	196	0.72	0.16	0.60	0.75	0.85	0.250
	G/C	313	0.71	0.16	0.60	0.75	0.80	
	C/C	160	0.77	0.14	0.66	0.80	0.85	

**RA**	G/G	232	1.21	0.38	0.95	1.24	1.47	**0.009**
	G/C	232	1.33	0.37	1.03	1.28	1.52	
	C/C	58	1.08^#^	0.47	0.73	1.10	1.41	

RNFL	G/G	231	0.20	0.14	0.13	0.18	0.25	0.519
	G/C	232	0.21	0.20	0.13	0.19	0.27	
	C/C	58	0.21	0.18	0.12	0.17	0.26	

NFI: nerve fiber index; c/d: cup disc ratio; RA: rim area; RNFL: retinal nerve fiber layer.

Tukey's HSD test: *P* < 0.05 (^#^G/C versus C/C).

**Table 7:** Analysis of *GRIN2B* (rs3764028) gene polymorphism depending on the clinical parameters in patients with primary open angle glaucoma (POAG) for each eye counted.

Clinical parameter	Genotype	POAG patients	Mean	SD	Quartile 25%	Median	Quartile 75%	*P*
NFI	C/C	364	29.9	19.8	16.0	25.0	37.0	0.339
	C/A	36	36.0	28.3	18.0	24.5	50.5	
	A/A	2	12.5	14.8	2.00	12.5	23.0	

c/d	C/C	609	0.72	0.16	0.60	0.75	0.85	0.124
	C/A	58	0.73	0.15	0.60	0.75	0.85	
	A/A	2	0.50	0	0.50	0.50	0.50	

RA	C/C	473	1.25	0.40	0.97	1.26	1.50	0.538
	C/A	47	1.18	0.36	0.92	1.23	1.43	
	A/A	2	1.34	0.02	1.32	1.34	1.35	

RNFL	C/C	472	0.20	0.17	0.12	0.19	0.26	0.434
	C/A	47	0.23	0.19	0.14	0.21	0.25	
	A/A	2	0.20	0.01	0.20	0.20	0.21	

NFI: nerve fiber index; c/d: cup disc ratio; RA: rim area; RNFL: retinal nerve fiber layer.

**Table 8:** Analysis of *APOE* (rs449647) gene polymorphism depending on the clinical parameters in patients with primary open angle glaucoma (POAG) for each eye counted.

Clinical parameter	Genotype	POAG patients	Mean	SD	Quartile 25%	Median	Quartile 75%	*P*
NFI	A/A	225	28.9	20.4	16.0	24.0	36.0	0.341
	A/T	146	31.7	20.7	17.0	26.0	43.0	
	T/T	31	32.9	23.5	18.3	26.0	38.5	

c/d	A/A	384	0.71	0.16	0.60	0.75	0.85	0.713
	A/T	227	0.72	0.16	0.60	0.70	0.85	
	T/T	58	0.75	0.11	0.70	0.80	0.80	

RA	A/A	298	1.24	0.39	0.95	1.24	1.48	0.620
	A/T	178	1.28	0.42	1.03	1.26	1.48	
	T/T	46	1.21	0.33	1.01	1.29	1.46	

RNFL	A/A	297	0.21	0.19	0.12	0.18	0.25	0.750
	A/T	178	0.20	0.16	0.14	0.19	0.26	
	T/T	46	0.20	0.08	0.15	0.18	0.26	

NFI: nerve fiber index; c/d: cup disc ratio; RA: rim area; RNFL: retinal nerve fiber layer.

**Table 9:** Comparison of mRNA expression in the blood of POAG with genotypes of genes. If *P* < 0.05 differences between each genotype and others tested *post hoc* with Tukey's HSD test.

Gene	Genotype	POAG patients	Mean	SD	Quartile 25%	Median	Quartile 75%	*P* ^(ANOVA)^
***HSP70-1***	G/G	20	2.77	0.85	2.12	2.68	3.62	**0.016**
	G/C	22	3.46^#^	0.78	0.78	3.02	3.92	
	C/C	6	3.51^∗^	0.64	3.32	3.47	3.52	

*BNDF *	C/C	9	19.84	1.38	19.07	19.47	21.25	0.183
	C/T	32	19.52	1.43	18.57	19.55	20.43	
	T/T	7	18.61	0.80	18.24	18.87	19.04	

*GRIN2B *	C/C	44	18.02	1.94	16.57	18.29	19.46	0.325
	C/A	6	18.92	0.83	18.52	18.76	19.43	
	A/A	—	—	—	—	—	—	

*APOE *	A/A	32	12.46	3.52	10.89	11.33	12.65	0.267
	A/T	15	11.20	0.93	10.48	10.89	11.94	
	T/T	1	12.59	—	12.59	12.59	12.59	

Tukey's HSD test: *P* < 0.05 (^∗^G/G versus C/C, ^#^G/G versus G/C).

**Table 10:** Analysis of *BDNF* expression level depending on the clinical parameters in patients with primary open angle glaucoma (POAG) for each eye counted.

Clinical parameter	Stage of POAG	Number of POAG	Mean (expression level − ΔCt)	SD (expression level − ΔCt)	Quartile 25%	Median	Quartile 75%	*P*
	Normal (0–30)	53	19.43	1.43	18.47	19.46	20.43	
NFI	Early (31–50)	13	19.34	1.03	18.89	19.50	19.84	0.681
	Advanced (>51)	4	18.82	0.77	18.28	18.75	19.36	

	Normal (<0.3)	0	—	—	—	—	—	
c/d	Early (0.3–0.7)	53	19.56	1.44	18.60	19.19	20.71	0.260
	Advanced (>0.7)	50	19.18	1.28	18.16	19.09	19.89	

**RA**	Normal (1.39–1.78)	33	20.33	1.25	19.06	19.67	20.65	**0.011**
	Early (1.26–1.38)	9	19.94	1.52	18.87	19.50	21.50	
	Middle-advanced (0.81–1.25)	34	19.25	1.16	18.87	19.28	19.82	
	Advanced (<0.81)	9	18.68^∧^	1.50	17.76	18.21	18.91	

RNFL	Normal (0.21–0.31)	39	19.79	1.34	18.94	19.62	20.59	0.360
	Early (0.20–0.18)	7	19.82	1.48	19.28	19.83	20.39	
	Middle-advanced (0.13–0.17)	18	19.17	1.05	18.60	19.26	19.77	
	Advanced (<0.13)	21	19.40	1.47	18.21	19.06	19.82	

NFI: nerve fiber index; c/d: cup disc ratio; RA: rim area; RNFL: retinal nerve fiber layer.

Tukey's HSD test: *P* < 0.05 (^∧^normal versus advanced).

**Table 11:** Analysis of *HSP70-1* expression level depending on the clinical parameters in patients with primary open angle glaucoma (POAG) for each eye counted.

Clinical parameter	Stage of POAG	Number of POAG	Mean (expression level − ΔCt)	SD (expression level − ΔCt)	Quartile 25%	Median	Quartile 75%	*P*
	Normal (0–30)	53	2.93	0.88	2.35	3.08	3.53	
NFI	Early (31–50)	13	3.04	0.92	2.21	3.46	3.64	0.814
	Advanced (>51)	4	2.73	0.62	2.49	2.76	2.99	

	Normal (<0.3)	0	—	—	—	—	—	
c/d	Early (0.3–0.7)	53	2.96	0.89	2.21	2.97	3.73	0.121
	Advanced (>0.7)	50	3.23	0.84	2.69	3.46	3.84	

**RA**	Normal (1.39–1.78)	33	3.07	0.93	2.35	3.38	3.83	**0.004**
	Early (1.26–1.38)	8	2.68	0.74	2.03	2.64	3.35	
	Middle-advanced (0.81–1.25)	34	2.85	0.85	2.21	3.01	3.53	
	Advanced (<0.81)	9	3.94^∧∗#^	0.48	3.58	3.89	4.27	

RNFL	Normal (0.21–0.31)	38	3.09	0.99	2.35	3.36	3.83	0.403
	Early (0.20–0.18)	7	2.78	0.69	2.22	2.61	3.41	
	Middle-advanced (0.13–0.17)	19	2.80	0.83	2.01	2.84	3.51	
	Advanced (<0.13)	21	3.18	0.88	2.59	3.46	3.85	

NFI: nerve fiber index; c/d: cup disc ratio; RA: rim area; RNFL: retinal nerve fiber layer.

Tukey's HSD test: *P* < 0.05 (^∧^normal versus advanced; ^∗^early versus advanced; ^#^middle-advanced versus advanced).

**Table 12:** Analysis of *GRIN2B* expression level depending on the clinical parameters in patients with primary open angle glaucoma (POAG) for each eye counted.

Clinical parameter	Stage of POAG	Number of POAG	Mean (expression level - ΔCt)	SD (expression level - ΔCt)	Quartile 25%	Median	Quartile 75%	*P*
	Normal (0–30)	53	17.99	2.04	16.59	18.26	19.47	
NFI	Early (31–50)	13	18.24	1.64	16.89	18.32	19.53	0.923
	Advanced (>51)	4	18.03	2.06	16.43	18.02	19.62	

	Normal (<0.3)	0	—	—	—	—	—	
c/d	Early (0.3–0.7)	53	18.02	1.70	16.78	18.32	19.43	0.614
	Advanced (>0.7)	50	18.20	1.98	16.64	18.46	19.56	

RA	Normal (1.39–1.78)	34	18.46	1.73	18.09	18.70	19.52	0.485
	Early (1.26–1.38)	8	18.02	1.16	17.35	18.09	18.54	
	Middle-advanced (0.81–1.25)	34	18.05	2.08	16.43	18.34	19.53	
	Advanced (<0.81)	9	17.51	1.29	16.59	17.46	18.45	

RNFL	Normal (0.21–0.31)	40	17.99	1.96	16.4	18.24	19.37	0.869
	Early (0.20–0.18)	6	18.16	1.88	17.32	18.65	19.36	
	Middle-advanced (0.13–0.17)	19	18.34	1.88	17.65	18.87	19.49	
	Advanced (<0.13)	20	18.34	1.43	16.80	18.49	19.53	

NFI: nerve fiber index; c/d: cup disc ratio; RA: rim area; RNFL: retinal nerve fiber layer.

**Table 13:** Analysis of *APOE* expression level depending on the clinical parameters in patients with primary open angle glaucoma (POAG) for each eye counted.

Clinical parameter	Stage of POAG	Number of POAG	Mean (expression level - ΔCt)	SD (expression level - ΔCt)	Quartile 25%	Median	Quartile 75%	*P*
	Normal (0–30)	53	12.55	3.88	10.42	11.32	12.68	
NFI	Early (31–50)	12	11.24	0.94	10.50	10.89	12.20	0.735
	Advanced (>51)	5	11.61	0.73	11.84	11.84	12.03	

	Normal (<0.3)	0	—	—	—	—	—	
c/d	Early (0.3–0.7)	53	12.35	3.89	10.46	11.11	12.58	0.805
	Advanced (>0.7)	50	12.09	2.91	10.87	11.12	11.98	

RA	Normal (1.39–1.78)	38	11.55	2.52	10.45	11.07	11.67	0.412
	Early (1.26–1.38)	6	14.92	6.27	10.76	11.67	19.39	
	Middle-advanced (0.81–1.25)	32	11.36	0.94	10.46	11.06	12.40	
	Advanced (<0.81)	9	11.53	0.39	11.24	11.68	11.86	

RNFL	Normal (0.21–0.31)	46	11.72	2.43	10.78	11.07	12.18	0.875
	Early (0.20–0.18)	6	11.51	1.01	10.46	11.49	12.58	
	Middle-advanced (0.13–0.17)	19	12.00	3.47	10.33	11.32	12.59	
	Advanced (<0.13)	20	11.51	1.98	10.56	11.22	11.60	

NFI: nerve fiber index; c/d: cup disc ratio; RA: rim area; RNFL: retinal nerve fiber layer.

## References

[1] Friedman D. S., Wolfs R. C., O'Colmain B. J. (2004). Prevalence of open-angle glaucoma among adults in the United States. *Archives of Ophthalmology*.

[2] Kama-Matyjaszek U., Sierzantowicz R., Mariak Z. (2010). Acceptance of own disease by patients with diagnosed glaucoma. *Polski Merkuriusz Lekarski*.

[3] Quigley H. A., Broman A. T. (2006). The number of people with glaucoma worldwide in 2010 and 2020. *British Journal of Ophthalmology*.

[4] Mozaffarieh M., Grieshaber M. C., Flammer J. (2008). Oxygen and blood flow: players in the pathogenesis of glaucoma. *Molecular Vision*.

[5] Distelhorst J. S., Hughes G. M. (2003). Open-angle glaucoma. *American Family Physician*.

[6] Gupta N., Yucel Y. H. (2007). Glaucoma as a neurodegenerative disease. *Current Opinion in Ophthalmology*.

[7] Bayer A. U., Keller O. N., Ferrari F., Maag K.-P. (2002). Association of glaucoma with neurodegenerative diseases with apoptotic cell death: alzheimer's disease and Parkinson's disease. *American Journal of Ophthalmology*.

[8] Sadun A. A., Bassi C. J. (1990). Optic nerve damage in Alzheimer's disease. *Ophthalmology*.

[9] Fisher J. B., Jacobs D. A., Markowitz C. E. (2006). Relation of visual function to retinal nerve fiber layer thickness in multiple sclerosis. *Ophthalmology*.

[10] Harwerth R. S., Vilupuru A. S., Rangaswamy N. V., Smith E. L. (2007). The relationship between nerve fiber layer and perimetry measurements. *Investigative Ophthalmology & Visual Science*.

[11] Nickells R. W. (1999). Apoptosis of retinal ganglion cells in glaucoma: an update of the molecular pathways involved in cell death. *Survey of Ophthalmology*.

[12] McKinnon S. J. (1997). Glaucoma, apoptosis, and neuroprotection. *Current Opinion in Ophthalmology*.

[13] Garcia-Valenzuela E., Shareef S., Walsh J., Sharma S. C. (1995). Programmed cell death of retinal ganglion cells during experimental glaucoma. *Experimental Eye Research*.

[14] Quigley H. A., Guy J., Anderson D. R. (1979). Blockade of rapid axonal transport. Effect of intraocular pressure elevation in primate optic nerve. *Archives of Ophthalmology*.

[15] Dreyer E. B., Zurakowski D., Schumer R. A., Podos S. M., Lipton S. A. (1996). Elevated glutamate levels in the vitreous body of humans and monkeys with glaucoma. *Archives of Ophthalmology*.

[16] Tezel G., Seigel G. M., Wax M. B. (1998). Autoantibodies to small heat shock proteins in glaucoma. *Investigative Ophthalmology and Visual Science*.

[17] Osborne N. N., Ugarte M., Chao M. (1999). Neuroprotection in relation to retinal ischemia and relevance to glaucoma. *Survey of Ophthalmology*.

[18] Kuehn M. H., Fingert J. H., Kwon Y. H. (2005). Retinal ganglion cell death in glaucoma: mechanisms and neuroprotective strategies. *Ophthalmology Clinics of North America*.

[19] Kaul M., Garden G. A., Lipton S. A. (2001). Pathways to neuronal injury and apoptosis in HIV-associated dementia. *Nature*.

[20] Tezel G., Hernandez M. R., Wax M. B. (2000). Immunostaining of heat shock proteins in the retina and optic nerve head of normal and glaucomatous eyes. *Archives of Ophthalmology*.

[21] Tezel G., Yang J., Wax M. B. (2004). Heat shock proteins, immunity and glaucoma. *Brain Research Bulletin*.

[22] Mahley R. W. (1988). Apolipoprotein E: cholesterol transport protein with expanding role in cell biology. *Science*.

[23] Herz J., Beffert U. (2000). Apolipoprotein E receptors: linking brain development and Alzheimer's disease. *Nature Reviews Neuroscience*.

[24] Poirier J. (2003). Apolipoprotein E and cholesterol metabolism in the pathogenesis and treatment of Alzheimer’s disease. *Trends in Molecular Medicine*.

[25] Wolozin B. (2004). Cholesterol and the biology of Alzheimer's disease. *Neuron*.

[26] Mann D. M. A., Iwatsubo T., Pickering-Brown S. M., Owen F., Saido T. C., Perry R. H. (1997). Preferential deposition of amyloid beta protein (Abeta) in the form Abeta40 in Alzheimer's disease is associated with a gene dosage effect of the apolipoprotein E E4 allele. *Neuroscience Letters*.

[27] Ayub H., Imran Khan M., Micheal S. (2010). Association of *eNOS* and *HSP70* gene polymorphisms with glaucoma in Pakistani cohorts. *Molecular Vision*.

[28] Jiang H. Q., Jia J. P. (2009). Association between NR2B subunit gene (*GRIN2B*) promoter polymorphisms and sporadic Alzheimer's disease in the North Chinese population. *Neuroscience Letters*.

[29] Wang J. C., Kwon J. M., Shah P., Morris J. C., Goate A. (2000). Effect of APOE genotype and promoter polymorphism on risk of Alzheimer's disease. *Neurology*.

[30] Artiga M. J., Bullido M. J., Sastre I. (1998). Allelic polymorphisms in the transcriptional regulatory region of apolipoprotein E gene. *FEBS Letters*.

[31] Dmitrzak-Weglarz M., Rybakowski J. K., Suwalska A. (2008). Association studies of the *BDNF* and the *NTRK2* gene polymorphisms with prophylactic lithium response in bipolar patients. *Pharmacogenomics*.

[32] Faul F., Erdfelder E., Lang A.-G., Buchner A. (2007). G∗Power 3: a flexible statistical power analysis program for the social, behavioral, and biomedical sciences. *Behavior Research Methods*.

[33] Mittag T. W., Danias J., Pohorenec G. (2000). Retinal damage after 3 to 4 months of elevated intraocular pressure in a rat glaucoma model. *Investigative Ophthalmology and Visual Science*.

[34] Kerrigan L. A., Zack D. J., Quigley H. A., Smith S. D., Pease M. E. (1997). TUNEL-positive ganglion cells in human primary open-angle glaucoma. *Archives of Ophthalmology*.

[35] Zhang S., Qian X., Redman C. (2003). p16^INK4a^ gene promoter variation and differential binding of a repressor, the ras-responsive zinc-finger transcription factor, RREB. *Oncogene*.

[36] Yang X., Martin T. A., Jiang W. G. (2012). Biological influence of brain-derived neurotrophic factor on breast cancer cells. *International Journal of Oncology*.

[37] Schuler M., Green D. R. (2001). Mechanisms of p53-dependent apoptosis. *Biochemical Society Transactions*.

[38] Kalita K., Makonchuk D., Gomes C., Zheng J.-J., Hetman M. (2008). Inhibition of nucleolar transcription as a trigger for neuronal apoptosis. *Journal of Neurochemistry*.

[39] Quigley H. A., McKinnon S. J., Zack D. J. (2000). Retrograde axonal transport of BDNF in retinal ganglion cells is blocked by acute IOP elevation in rats. *Investigative Ophthalmology and Visual Science*.

[40] Pease M. E., McKinnon S. J., Quigley H. A., Kerrigan-Baumrind L. A., Zack D. J. (2000). Obstructed axonal transport of BDNF and its receptor TrkB in experimental glaucoma. *Investigative Ophthalmology & Visual Science*.

[41] Gupta V., You Y., Li J. (2014). BDNF impairment is associated with age-related changes in the inner retina and exacerbates experimental glaucoma. *Biochimica et Biophysica Acta*.

[42] Gao H., Qiao X., Hefti F., Hollyfield J. G., Knusel B. (1997). Elevated mRNA expression of brain-derived neurotrophic factor in retinal ganglion cell layer after optic nerve injury. *Investigative Ophthalmology & Visual Science*.

[43] Vepsäläinen S., Castren E., Helisalmi S. (2005). Genetic analysis of BDNF and TrkB gene polymorphisms in Alzheimer’s disease. *Journal of Neurology*.

[44] Pae C.-U., Chiesa A., Porcelli S. (2012). Influence of *BDNF* variants on diagnosis and response to treatment in patients with major depression, bipolar disorder and schizophrenia. *Neuropsychobiology*.

[45] Chidlow G., Wood J. P. M., Casson R. J. (2014). Expression of inducible heat shock proteins Hsp27 and Hsp70 in the visual pathway of rats subjected to various models of retinal ganglion cell injury. *PLoS ONE*.

[46] Kwong J. M. K., Gu L., Nassiri N. (2014). AAV-mediated and pharmacological induction of Hsp70 expression stimulates survival of retinal ganglion cells following axonal injury. *Gene Therapy*.

[47] He M., Guo H., Yang X. (2009). Functional SNPs in *HSPA1A* gene predict risk of coronary heart disease. *PLoS ONE*.

[48] Tosaka K., Mashima Y., Funayama T., Ohtake Y. (2007). Association between open-angle glaucoma and gene polymorphism for heat-shock protein 70-1. *Japanese Journal of Ophthalmology*.

[49] Amaratunga A., Abraham C. R., Edwards R. B., Sandell J. H., Schreiber B. M., Fine R. E. (1996). Apolipoprotein E is synthesized in the retina by Müller glial cells, secreted into the vitreous, and rapidly transported into the optic nerve by retinal ganglion cells. *Journal of Biological Chemistry*.

[50] Vickers J. C., Craig J. E., Stankovich J. (2002). The apolipoprotein epsilon4 gene is associated with elevated risk of normal tension glaucoma. *Molecular Vision*.

[51] McKinnon S. J., Lehman D. M., Kerrigan-Baumrind L. A. (2002). Caspase activation and amyloid precursor protein cleavage in rat ocular hypertension. *Investigative Ophthalmology & Visual Science*.

[52] Copin B., Brézin A. P., Valtot F., Dascotte J.-C., Béchetoille A., Garchon H.-J. (2002). Apolipoprotein E-promoter single-nucleotide polymorphisms affect the phenotype of primary open-angle glaucoma and demonstrate interaction with the myocilin gene. *The American Journal of Human Genetics*.

